# Elevated CO_2_ Levels do not Affect the Shell Structure of the Bivalve *Arctica islandica* from the Western Baltic

**DOI:** 10.1371/journal.pone.0070106

**Published:** 2013-07-29

**Authors:** Kristina Stemmer, Gernot Nehrke, Thomas Brey

**Affiliations:** 1 Functional Ecology, Alfred Wegener Institute for Polar and Marine Research in the Helmholtz Association, Bremerhaven, Germany; 2 BioGeoScience, Alfred Wegener Institute for Polar and Marine Research in the Helmholtz Association, Bremerhaven, Germany; Texas A&M University, United States of America

## Abstract

Shells of the bivalve *Arctica islandica* are used to reconstruct paleo-environmental conditions (e.g. temperature) via biogeochemical proxies, i.e. biogenic components that are related closely to environmental parameters at the time of shell formation. Several studies have shown that proxies like element and isotope-ratios can be affected by shell growth and microstructure. Thus it is essential to evaluate the impact of changing environmental parameters such as high *p*CO_2_ and consequent changes in carbonate chemistry on shell properties to validate these biogeochemical proxies for a wider range of environmental conditions. Growth experiments with *Arctica islandica* from the Western Baltic Sea kept under different *p*CO_2_ levels (from 380 to 1120 µatm) indicate no affect of elevated *p*CO_2_ on shell growth or crystal microstructure, indicating that *A. islandica* shows an adaptation to a wider range of *p*CO_2_ levels than reported for other species. Accordingly, proxy information derived from *A. islandica* shells of this region contains no *p*CO_2_ related bias.

## Introduction

Marine biogenic carbonates such as bivalve shells represent complex composites of organic and inorganic phases [1], [2], [3]. Fossil and recent shells are valuable bioarchives for paleo-climate reconstructions [4], [5], [6] and environmental monitoring purposes [7]. Furthermore, bivalve shells provide information on environmental conditions at times of shell formation in the form of structural and biogeochemical properties [8], [9], [10]. However, in bivalve shells, some of the “classic” proxy systems (e.g. trace elements) developed for paleo-temperature, salinity and food availability have been shown to be affected by growth patterns, crystal fabric structure [11], the organic and the mineral phase of the biogenic carbonate (calcite, the more soluble aragonite or both) [11], [12], [13].

Increased seawater *p*CO_2_ and therefore decreased pH leads to a reduced saturation level for calcium carbonates [14] and therefore hampers shell or skeleton formation [15], [16]. Apparently, several species of marine calcifiers can cope with such conditions [16], [17], [18], [19] albeit this adaptation may coincide with changes in shell microstructure and chemistry [18], [19], [20], [21]. Raising atmospheric CO_2_ and the corresponding decrease in ocean pH represents a challenge for marine calcifiers on a global scale (e.g. [22]).

A number of studies evaluated the impact of high *p*CO_2_ and low pH on marine bivalve shells (e.g. [15], [16], [23], [24]) but just a few of these took a closer look on shell growth in height or thickness and on internal shell crystal fabrics [18], [20], [21]. In the North Atlantic bivalve *A. islandica*, Hiebenthal et al. [25] found shell stability, shell growth and tissue lipofuscin accumulation (indicating stress levels) to be unaffected by high *p*CO_2_ (up to 1700 µatm), indicating that this species may be less vulnerable to ocean acidification.

Due to its longevity (up to several centuries, [26], [27], [28]), its distinct internal growth band pattern [29], [30], its wide distribution in the northern Atlantic [31], [32], and its long fossil record *A. islandica* represents a valuable bioarchive [33], [34], [35]. Wanamaker et al. [36] e.g. used shell-derived temperature proxies (δ^18^O_c_) of *A. islandica* to reconstruct ocean temperature variability over the last millennium.

The mineral phase present within the shell of *A. islandica* is aragonite with an outer shell layer (OSL) comprising the outer shell margin and forming the distinct shell increments and growth checks, and an inner shell layer (ISL) extending from the oldest part of the shell, the umbo, to the pallial line [37], [38], [39]. Both layers are separated by a thin myostracum. In addition, a protective organic layer, the periostracum, covers the outer shell. The shell is formed at the inner shell surface (growth in thickness) and the shell margin (growth in height), i.e. at two separate locations of precipitation divided by the attachment of the mantle tissue at the pallial line: The inner extrapallial fluid (EPF) is in contact with the ISL that is not yet formed outward the pallial line where the outer EPF is in contact with the OSL. It is suggested that shell precipitates directly from the EPF situated in space between secretory mantle tissue and shell surface [40], [41], [42]. However, to what extent the EPF is involved in shell formation is not clear and subject of current research. Outer and inner shell layer are composed of distinct crystal morphotypes that can be differentiated in shell cross-sections, with different affinities to the uptake of elements [11], . Irregular simple prisms, irregular complex crossed lamellar and crossed acicular-crossed lamellar microstructures have been described by Ropes et al. [39] and are also observed in *A. islandica* shells from the North Sea [11], whereas the shells from the Kiel Bight (Western Baltic Sea) mostly display homogeneous crystals in the outer shell layer and simple crossed lamellar structures in the inner shell layer [37].

There is substantial evidence that element and isotope signatures of biogenic carbonates used as proxy data are affected by the crystal fabric structure of the biogenic carbonate to some extent [43], [44]. Crystal growth rate, size and crystal fabric structure within bivalve shells can strongly influence trace element concentrations, as shown by, e.g., Carré et al. [45]; Freitas et al. [46]. Regarding *A. islandica*, Schoene et al. [11] recommends to restrict sampling for geochemical analysis to one type of shell crystal fabric to avoid structure related bias.

In Kiel Bight, *Arctica islandica* lives below the thermohaline pycnocline (>15 m), and is thus exposed to strong environmental fluctuations, i.e. low and variable salinity (18–23), periods of low oxygen availability during summer stratification and correspondingly, fluctuating *p*CO_2_ levels with peaks over 1000 µatm (Boknis Eck Time Series Station, [47]. Shell growth and shell crystal fabric structure represent an integrated response of the physiological and biochemical activities in the organism to the surrounding environmental conditions [48]. Compared to *A. islandica* from fully marine environments, the life span of Kiel Bight animals is distinctly shorter, the shells are generally thinner [32] and smaller and show a less organized microstructure [37] than *A. islandica* from the North Sea or Iceland. Nevertheless, *A. islandica* is a prominent and abundant key species in Western Baltic benthic communities [49].

The aim of this study is to investigate the impact of *p*CO_2_ on the shell microstructure of *A. islandica* from the Kiel Bight, in order to evaluate the possible impact such changes would have on shell based proxies.

## Materials and Methods

### Ethics Statement

Field sampling did not require specific permissions but was in accordance with general governmental regulations. No endangered or protected species were involved.

### Aquaculture

Specimens of *Arctica islandica* were collected in February 2010 from the “Süderfahrt” location (N 54°31′–32′ E 10°41′–48′) in Kiel Bight, Western Baltic. Samples were dredged from the seafloor in 20 m water depth. Quahogs were transported to the AWI Wadden Sea Station Sylt and kept in an aerated flow-through tank with natural sediment for an acclimation time of 3 months. *A. islandica* is a high saline species and shows optimum growth at between 6 and 10°C [25], [50], [51]. Therefore, salinity and temperature of experimental seawater were slowly increased to experimental starting conditions with salinity of 29 and a temperature of 10°C. Small animals of 15 to 25 mm height were chosen for this study. The ontogenetic age of *A. islandica* from Kiel Bight in that size range (15–25 mm) is about 4–5 years [49], [52]. According to the *A. islandica* growth model of Begum et al. [52], shell production for the size range 15–25 mm amounts to 0.18 to 0.46 g calcium carbonate (CaCO_3_) y^−1^. This seems to be a comparatively narrow range compared to lifetime range in shell production (up to 1.27 g y^−1^). Shell sizes were distributed randomly over treatments. Initial shell size did not differ significantly between treatments (ANOVA, p = 0.288).

During the experiments, artificial calcium carbonate free sediment (Vitakraft® quarz gravel 1−2 mm grain size) was used to avoid pH buffering. Food supply (DT’s Premium Blend, T’s Plankton Farm, Sycamore, IL, containing *Nannochloropsis oculata*, *Phaeodactylum tricorutum* and *Chrorella* sp.), 3 ml was added every two days.

### Calcein Staining

To mark the start of the experiment the animals were immersed for 4½ hours in a calcein solution (100 mg/l seawater) followed by two washing steps as described in Riascos et al. [53]. Calcein is a fluorescent dye with an excitation and emission wavelength of 495/515 nm respectively. It is incorporated in biogenic calcium carbonate at the actual location of carbonate growth [54], i.e. the outer shell margin in bivalves [53] and was shown not to alter the element signature of the carbonate [55]. All clams were pumping water (shell open and siphons visible) during the staining period and were therefore exposed to the fluorochrome. No animal died during the staining. The perturbation experiment started immediately after the staining procedure.

### CO_2_ Perturbation Experiment

*A. islandica* were kept at three different *p*CO_2_ - gas-levels for 90 days: The control group at 380 µatm (ambient atmospheric CO_2_ level) and experimental groups at 760 µatm (2x actual *p*CO_2_) and 1120 µatm (4x preindustrial *p*CO_2_), respectively. CO_2_ concentrations of the experimental water were maintained by a gas mixing system (HTK, Hamburg, Germany). Experimental temperature was set to 10°C but showed slight fluctuations over the 90 days owing to North Sea warming as well as slight differences between incubations related to technical conditions (Table 1).

**Table 1:** Carbonate system parameters of the experimental seawater over time (90 days).

Measured parameters								
*p*CO_2_-gas [µatm]	A*_T_* [mmol kg^−1^]	SD	Sal	SD	T (°C)	SD	pH_NBS_	SD
380	2328	(±28)	30.9	(±0.8)	10.6	(±2.1)	8.07	(±0.05)
760	2335	(±26)	30.9	(±0.8)	9.5	(±2.1)	7.90	(±0.07)
1120	2335	(±26)	30.9	(±0.8)	9.3	(±1.3)	7.75	(±0.07)
**Calculated parameters**								
**C** ***_T_*** ** [mmol kg^−1^]**	**SD**	***p*** **CO_2_-sw [µatm]**	**SD**	**Ω arag**	**SD**			
2193	(±48)	524	(±83)	1.68	(±0.30)			
2263	(±45)	800	(±184)	1.14	(±0.26)			
2309	(±45)	1140	(±221)	0.83	(±0.18)			

Measured and calculated mean values. *p*CO_2_-gas [µatm] = concentration of *p*CO_2_ in perturbation gas; A*_T_* [mmol kg**^−^** ^1^] = Total Alkalinity; Sal = Salinity; T (°C) = Temperature in Celsius; pH_NBS_ = pH calibrated with Nist Buffer Standard; C*_T_* [mmol kg**^−^** ^1^] = Total dissolved inorganic carbon; *p*CO_2_-sw [µatm] = concentration of *p*CO_2_ in seawater; Ω arag = saturation state of aragonite; SD = standard deviation.

**Figure 1:**
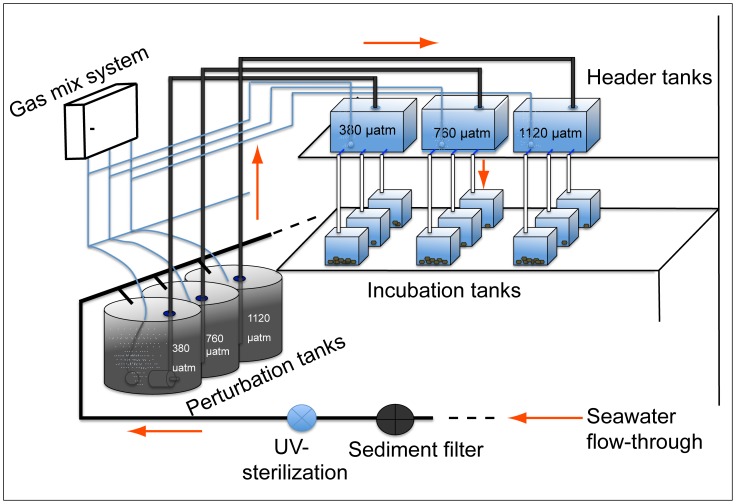
Experimental-setup for *p*CO_2_ perturbation. The temperature controlled room was supplied with one filter-tank (sediment filter and UV-sterilization), one 250 l perturbation tank and one 30 l perturbed header tank per *p*CO_2_-level. From the header tanks the CO_2_-enriched water and the control water flowed down to triplicates of 4 l incubation tanks. In each tank 10 animals were incubated, i.e. a total of 30 clams per *p*CO_2_-level. A plastic lid sealed all tanks to prevent gas exchange with the atmosphere. Seawater-flow rate from header to incubation tank was 150 ml/min.

The experimental setup is shown in Figure 1.

### Water Chemistry and Calculations

pH, salinity and temperature of the treatment tanks were measured once a day. To determine the total alkalinity, water samples (25 ml) (A_T_, determined by means of potentiometric titration using the Gran method) were collected once a week. The pH electrode (WTW 3310 pH meter with SenTix Mic electrode, Weilheim, Germany) was calibrated with NBS buffers before each measurement. Carbonate chemistry was calculated using the program CO2SYS [56] with the input of pH (NBS scale), A_T_ and the constants of Mehrbach et al. [57]. Measured and calculated water parameters from our controlled perturbation experiment under different *p*CO_2_ conditions are listed in Table 1.

### Shell Material

After 90 days experimental exposition, the quahogs were chucked and the soft tissues removed. Shells were carefully cleaned by hand and air-dried. From each treatment, 15 shells (5 per replicate) were randomly chosen for growth analysis. The staining with the fluorochrome calcein marked the start of the *p*CO_2_ incubation and allowed to identify shell material grown under experimental conditions.

### Growth Analysis

**Figure 2:**
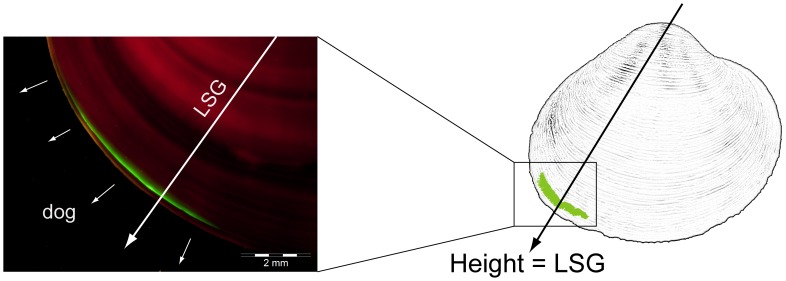
*Arctica islandica* shell showing a green calcein mark that indicates the start of the *p*CO_2_ incubation. New grown shell was measured at the line of strongest growth (LSG). Arrows indicate the direction of growth (dog).

Shells were submerged in NaOCl (13%) solution for 1 h to remove the organic layer (periostracum) and subsequently rinsed twice with de-ionisized water. Shells were checked for calcein marks using a fluorescence stereoscope (Olympus SZX12, Figure 2). In most shells, the calcein mark was not found along the whole shell edge, but only intermittently. If the mark was detectable at the line of strongest growth (LSG, Fig. 1), the shell was cross-sectioned along the LSG, if not, along a line through the calcein mark closest to the LSG. To prevent shell damage during cutting, metal epoxy (Toolcraft) was applied to the marked shell area one day before sectioning. Cross-sections were ground using grinding paper (Buehler) with grit sizes of P1200/P2400/P4000 followed by a polishing step with Buehler diamond polycrystalline suspensions (3 µm) and a final polish with aluminum oxide suspension (1 µm). The samples were then carefully rinsed using de-ionized water and air-dried.

**Figure 3:**
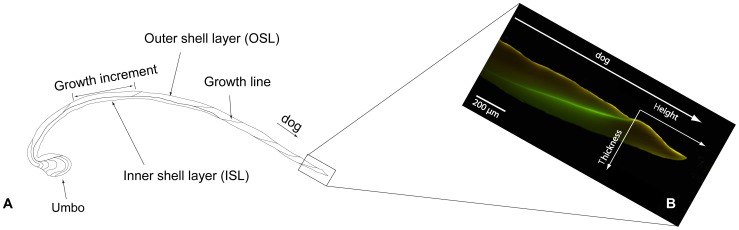
Shell growth measurements. (A) Sketch of shell-cross-section with major shell structures. (B) Magnification of outer shell margin from the cross-section showing calcein mark and additional shell growth in height and thickness.

Shell growth in height, i.e. addition of incremental growth, was measured from the end of the calcein mark (start of *p*CO_2_ incubation) to the outer shell margin (end of *p*CO_2_ incubation). If growth could not be measured directly on the LSG trajectory, the measurement was transformed to growth at LSG assuming isometric shell growth in all directions. Shell growth in thickness (i.e. the distance between inner and outer valve surface) was measured at the end of the calcein mark perpendicular to the direction of growth (Figure 3). All measurements were performed under a fluorescence stereoscope (Olympus SZX12) using the program ANALYSIS.

Differences in shell growth in height and thickness between treatments were analyzed by one-way ANOVA and subsequent TUKEY HSD post-hoc tests (significance level alpha = 0.05).

### Structural Analysis

**Figure 4:**
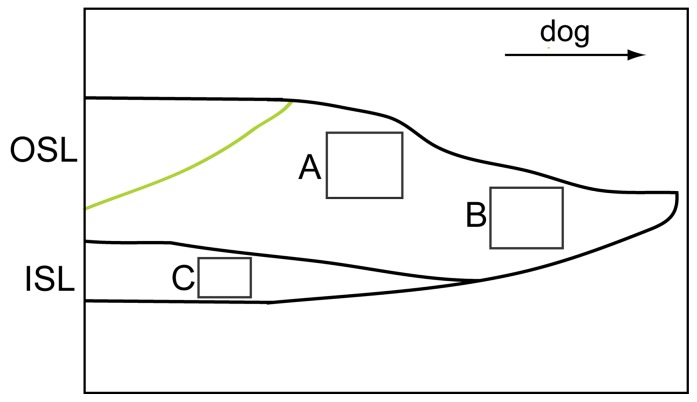
Sketch of outer shell margin from the cross section with areas where SEM images were taken. (A) Transition zone of shell material grown under normal and *p*CO_2_ perturbated conditions in the outer shell layer (OSL) (green line = calcein mark). (B) Inner shell layer (ISL). (C) Last precipitated shell material at the very tip of the shell.

Shell microstructures were studied by scanning electron microscopy (XL30 ESEM, Philips) of shell cross-sections. Polished samples were coated with gold and scanned with an accelerating Voltage of 10 kV and a beam current of 1.7 nA. Shell-layers chosen for analysis are shown in Figure 4 A, B, C.

## Results

### Shell Staining

The calcein mark used to mark the start of the experiment could be detected in 80% of the shells (Table 2) etched with NaOCl. Within most shells the calcein mark did not appear along the whole shell margin but only in the fastest growing segments (Figure 2). This indicates asynchronous shell growth of *Arctica islandica* during short time periods (4½ h calcein immersion). However, since this finding is outside the scope of this study we will not pursue it further here.

**Table 2:** Daily growth rates of *Arctica islandica*.

No.	sample	*p*CO_2_ [µatm]	calcein mark	shell growth at LSG	
				in height [µm/day]	in thickness [µm/day]
1	380A1	380	×	3.10	1.78
2	380A2	380	×	1.18	0.88
3	380A3	380	×	3.01	2.02
4	380A4	380	×	3.64	2.06
5	380A5	380	–	–	–
6	380B1	380	×	2.35	2.88
7	380B2	380	×	2.05	1.34
8	380B3	380	×	2.33	1.79
9	380B4	380	–	–	–
10	380B5	380	–	–	–
11	380C1	380	×	1.32	1.29
12	380C2	380	×	2.38	1.21
13	380C3	380	×	1.56	1.00
14	380C4	380	×	4.55	1.99
15	380C5	380	–	–	–
16	760A1	760	×	1.51	1.27
17	760A2	760	×	2.01	1.53
18	760A3	760	×	1.35	1.27
19	760A4	760	×	7.04	2.38
20	760A5	760	–	–	–
21	760B1	760	×	0.98	0.89
22	760B2	760	×	1.14	0.84
23	760B3	760	×	1.09	1.18
24	760B4	760	–	–	–
25	760B5	760	–	–	–
26	760C1	760	×	1.84	1.49
27	760C2	760	×	3.76	1.72
28	760C3	760	×	1.86	1.09
29	760C4	760	×	2.24	1.70
30	760C5	760	×	1.77	1.09
31	1120A1	1120	×	2.57	1.58
32	1120A2	1120	×	1.78	0.95
33	1120A3	1120	×	1.31	0.97
34	1120A4	1120	×	2.15	1.43
35	1120A5	1120	×	1.75	1.07
36	1120B1	1120	×	3.54	2.40
37	1120B2	1120	×	0.49	0.70
38	1120B3	1120	×	1.66	1.37
39	1120B4	1120	–	–	–
40	1120B5	1120	–	–	–
41	1120C1	1120	×	1.86	1.37
42	1120C2	1120	×	4.11	1.82
43	1120C3	1120	×	1.32	1.77
44	1120C4	1120	×	2.09	1.46
45	1120C5	1120	×	2.99	1.26

Column five and six give the shell growth rate per day at the line of strongest growth (LSG) in height and in thickness. To present shell growth rate per day, the measured total shell growth during the experiment was divided by the 90 days of the experiment. No. = continuous sample number; sample = sample code; *p*CO_2_ [µatm] = concentration of gas-mix applied.

### Growth Rate

**Figure 5:**
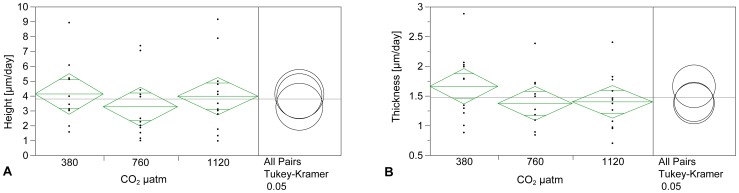
Shell growth in height (A) and thickness (B) did not differ significantly under three different *p*CO_2_ levels (n = 11–13, one-way ANOVA, height: = 0.503, p = 0.609; thickness: F = 1.227, p = 0.306; JMP9).

*p*CO_2_ level had no significant effect on shell growth in height and thickness (one-way ANOVA, height: F = 0.503, p = 0.609; thickness: F = 1.227, p = 0.306). Growth varied between 0.96 µm/day and 9.14 µm/day in height and between 0.70 µm/day and 2.88 µm/day in thickness (Figure 5A, B respectively; Table 2).

### Shell Microstructure

**Figure 6:**
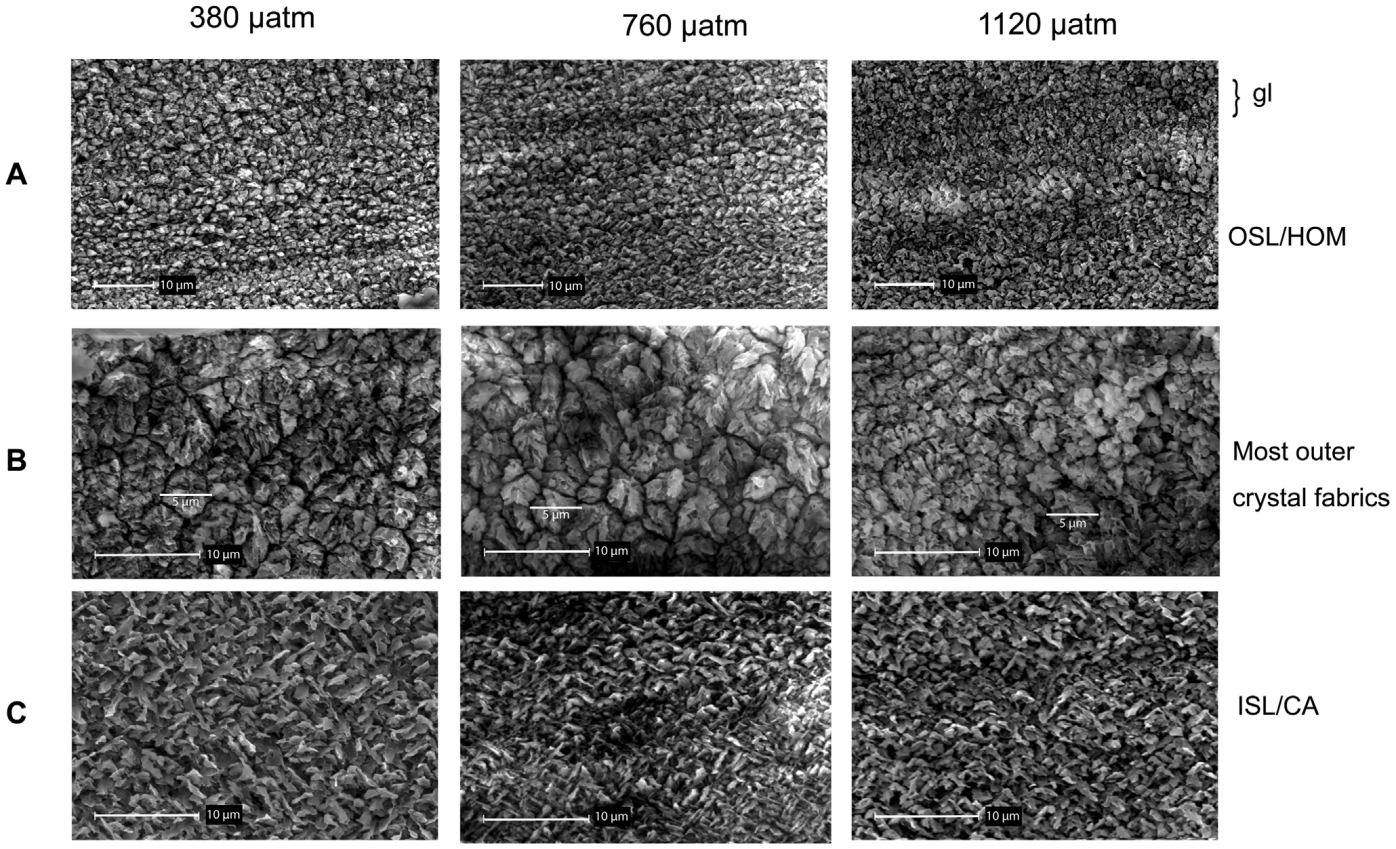
SEM images. Crystal fabrics of new shell material grown under different *p*CO_2_ levels. (A) The growth line (gl) stained with calcein at starter point of the *p*CO_2_ incubation is visible due to smaller crystals. HOM = homogeneous crystal fabric. (B) Inner shell layer (ISL) with distinct crossed-acicular crystal fabric. (C) Latest formed crystal fabric.

Shell crystal fabrics of *A. islandica* formed during the *p*CO_2_ incubations did not differ between exposures to *p*CO_2_ levels ranging from 380 to 1120 µatm (Figure 6). In the cross-sections, the outer and inner shell layers (OSL/ISL) were clearly distinguishable by their crystal fabrics: The shell region of the OSL is characterized by a homogeneous distribution of irregular shaped crystals with an average diameter of 1.5 µm (Figure 6A). The inner shell layer was build from distinct crossed-lamellar crystal fabrics [11], [37] (Figure 4B; Figure 6B). The tips of the very recently formed shell, i.e. the latest formed crystal fabrics are also similar in all experimental animals: They consist of homogeneously distributed but irregularly shaped crystals with an average diameter of 5 µm (Figure 4C; Figure 6C).

## Discussion

Our study indicates that shell growth and crystal microstructure of *Arctica islandica* from Kiel Bight are not altered by *p*CO_2_ in the range of 380–1120 µatm.

Individual shell growth rates varied over a wide range (0.96–9.14 µm/day in height and 0.70–2.88 µm/day in thickness) but were not affected by the *p*CO_2_ treatment. Each shell formed new shell material with distinct and specific crystal fabrics in the outer and inner shell layers. The widely accepted concept of extracellular matrix mediated mineralization in bivalves [1], [58] suggests that mineral formation requires a microenvironment that provides and maintains a sufficient supersaturation for nucleation and growth of the mineral phase. Our results indicate that *A. islandica* possesses a high physiological control over the chemical composition at the site of calcification, even when exposed to elevated proton concentrations, i.e. low pH.

Current implications suggest that elevated *p*CO_2_ and lowered pH can have various effects on bivalve species and other marine calcifiers, apparently depending on species and experimental conditions [15], [20], [21], [23], [59]. Accordingly, we are still far from a consistent picture of the cause-and-effect mechanisms involved.

To our knowledge there are few studies of *p*CO_2_ effects on newly grown bivalve shell material. Hahn et al. [20] report changes in shell ultrastructure of the Mediterranean *Mytilus galloprovincialis* that were transplanted in the field from normal to a high *p*CO_2_ level site with a pH of 8.1 and 7.3 respectively. However, it remains unclear to which extent other (uncontrolled) environmental factors may have also affected shell properties. Melzner et al. [21] observed dissolution of the internal aragonite (nacre) layer in the blue mussel *Mytilus edulis* from Kiel Fjord under exposition to *p*CO_2_>2000 µatm. These mussels experience seasonal *p*CO_2_ peaks of >4000 µatm and corresponding pH values as low as 7.1 and thus are presumed to be adapted to such conditions [18], [25]. However, a direct comparison with our findings is difficult because (i) the shell of *M. edulis* consists of two calcium carbonate polymorphs, calcite on the outside and aragonite on the inside and (ii) *p*CO_2_ impact on newly grown shell material in shell regions comparable to those investigated in our study was not analyzed.

### What makes Arctica Islandica so Special?

The findings that elevated *p*CO_2_ levels neither affect shell growth rate ([25], this study) nor shell microstructure (this study) indicate that *A. islandica* is in full physiological and chemical control of the shell formation process, including carbonate precipitation. This tolerance can have two possible explanations: (a) pre-adaptation through species-specific lifestyle; (b) pre-adaptation to regularly enhanced *p*CO_2_ levels in Kiel Bight.

- *A. islandica* is unique among bivalves as the deliberate exposition to high *p*CO_2_ and low pH conditions is part of its life strategy: *A. islandica* can perform extreme “metabolic rate depression” (MDR), i.e. animals may stop water pumping and bury deeper into the sediment for several days, while reducing metabolic activity to very low levels [26], [60]. These sediments are often hypercapnic (physiological effects of elevated *p*CO_2_) and can be undersaturated with respect to aragonite [61], whereas body fluids, i.e. haemolymph, mantle water, and extrapallial fluid are naturally acidified and may become even more acidic under anaerobic conditions [62], [63]. Our findings support the hypothesis of Hiebenthal et al. [25] that the specific lifestyle of *A. islandica* may serve as a pre-adaptation to forthcoming elevated ocean *p*CO_2_. This feature may have also added to the long-term success of *A. islandica*. *A. islandica* is the only remnant of an ancient genus of the once diverse Arcticidae [32] and apparently was able to survive major past climatic oscillations, showing a high abundance through geological times and a wide distribution in the Northern Atlantic. Presence of the genus *Arctica* during the high CO_2_ Cretaceous epoch (3.7 to 14.7 times the modern pre-industrial value of 285 ppm) [64], [65] indicates that this species may be pre-adapted to high *p*CO_2_ levels in general and not only in a Baltic sub-population.
- An alternative explanation would be that *A. islandica* from Kiel Bight are well adapted to the strongly fluctuating conditions (salinity, temperature, oxygen availability, *p*CO_2_) at this locality. As a consequence this population can tolerate also elevated *p*CO_2_ levels. Adaption to fluctuating and increased *p*CO_2_ may add to the general expression of pronounced stress response at the expense of lifespan [66] with that of Kiel Bight animals of ∼40 yrs [67] compared to *A. islandica* from Iceland populations living up to 400 yrs in fully marine environment [34], [36]. The robustness towards changing conditions of Kiel Bight animals is also reflected in our experiment where *A. islandica* was not affected by fully saline North Sea water used during the experiment.

Future research on *A. islandica* from different localities (Iceland, Kattegat, White Sea, ect.), possibly in combination with genetic approaches (e.g. transcriptomics) [66] will show whether or not the observed *p*CO_2_ tolerance of this bivalve is unique for the Kiel Bight population or if represents a species-specific feature.

However, synergistic effects of *p*CO_2_ and other parameters such as temperature, food availability and salinity have not been considered yet. Furthermore, we still lack a detailed understanding of the mechanisms and controls of shell formation, which are a matter of ongoing and future research. We need to uncover the processes involved in biomineralization and before resolving this interdisciplinary enigma we can only report species-specific responses and hypothesize the processes behind it.

### Conclusions

Our study shows that shell growth and shell microstructure of young *Arctica islandica* from Kiel Bight are not affected by the *p*CO_2_ up to 1120 µatm. Correspondingly, isotope and element-based proxies derived from *A. islandica* shells are unbiased regarding changes in shell crystal fabric structure caused by varying environmental *p*CO_2_ levels. Whether or not this robustness applies to all *A. islandica* populations or just to the one from the Western Baltic remains to be seen.
